# Ataxia and developmental delay as the main manifestation of rhombencephalosynapsis

**Published:** 2018-01-05

**Authors:** Mohammad Paktinat, Soroor Inaloo, Zahra Serati, Eslam Shorafa

**Affiliations:** Department of Pediatrics, School of Medicine, Shiraz University of Medical Sciences, Shiraz, Iran

**Keywords:** Cerebellum Diseases, Cerebellar Vermis, Ataxia, Developmental Delay

A 2.5-year-old boy born of unrelated parents, without abnormal perinatal history, term, product of normal vaginal delivery, without history of hospitalization, presented with abnormal gait and developmental delay. He started to walk by 18 months and speak just few words. By now, he has normal weight gain. There was no family history of neurological illness. On examination, he had no dysmorphic feature, normal cranial nerves, and normal tendon reflexes. His gait was ataxic.

His brain magnetic resonance imaging revealed fusion of the cerebellar hemispheres with agenesis of vermis suggesting rhombencephalosynapsis (RS) (Figure 1). There were no abnormalities of the supratentorial structures.

RS is an congenital pathologic condition with broad spectrum of clinical and imaging manifestations and with sporadic prevalence. 

It is associated with posterior fossa malformation that defined by hypogenesis or agenesis of the vermis, dorsal fusion of the cerebellar hemispheres, and fusion of the dentate nuclei and superior cerebellar peduncles.^1^ Approximately, 100 cases of RS are reported in literature.^2^ RS is frequently described in association with Gómez-López-Hernández syndrome, also may occurs in conjunction with vertebral defects, anal atresia, cardiac defects, tracheo-esophageal fistula, renal anomalies, and limb abnormalities (VACTERL features) and with holoprosencephaly.^3^

**Figure 1:**
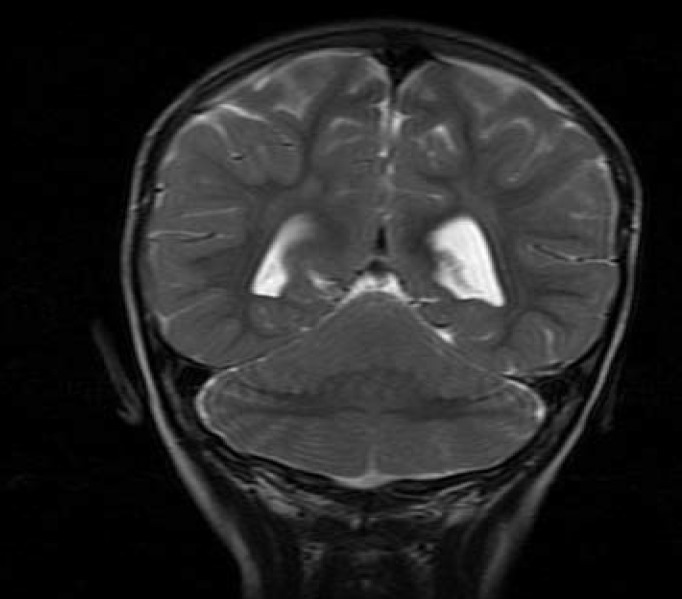
Fusion of the cerebellar hemispheres with agenesis of vermis

Clinical and imaging findings include persistent head-shaking (in about 85% of cases),^4^ muscular hypotonia, spasticity, head rolling, abnormal eye movement, strabismus, dysarthria, poor balance, seizure, mental retardation, attention deficit, cognitive impairment, psychiatric disorders, and developmental delay.^5^ According to our case report, it should be suspected in the presence of ataxia and abnormal gait with other aspects of developmental abnormality.

## Notes:

**How to cite this article:** Paktinat M, Inaloo S, Serati Z, Shorafa E. Ataxia and developmental delay as the main manifestation of rhombencephalosynapsis. Iran J Neurol 2018; 17(1): 56-7.
